# Patients With Inflammatory Bowel Disease Are at Increased Risk of Respiratory Syncytial Virus Infections After Severe Acute Respiratory Syndrome Coronavirus 2 Infection: A Propensity-Matched Cohort Analysis

**DOI:** 10.14309/ctg.0000000000000840

**Published:** 2025-03-27

**Authors:** Saqr Alsakarneh, Oscar Ramirez Ramirez, Mary S. Hayney, Jana G. Hashash, Francis A. Farraye, Freddy Caldera

**Affiliations:** 1 Department of Medicine, University of Missouri-Kansas City, Kansas City, Missouri, USA;; 2 Department Medicine, Division of Internal Medicine, University of Wisconsin-Madison School of Medicine & Public Health, Madison, Wisconsin, USA;; 3 School of Pharmacy, School of Medicine & Public Health, University of Wisconsin--Madison, Madison, Wisconsin, USA;; 4 Department of Gastroenterology and Hepatology, Inflammatory Bowel Disease Center, Mayo Clinic, Jacksonville, Florida, USA;; 5 Department of Medicine, Division of Gastroenterology and Hepatology, School of Medicine & Public Health, University of Wisconsin—Madison, Madison, Wisconsin, USA.

**Keywords:** inflammatory bowel disease, COVID-19, immunosuppression, respiratory infection

## Abstract

**INTRODUCTION:**

Patients with inflammatory bowel disease (IBD) are at an increased risk of infections. Before the COVID-19 pandemic, respiratory syncytial virus (RSV) followed predictable seasonal patterns, which have been recently disrupted. This study aimed to investigate whether severe acute respiratory syndrome (SARS) coronavirus 2 (CoV-2) infection is associated with an increased risk of RSV infection in patients with IBD compared with those without a history of SARS-CoV-2 infection.

**METHODS:**

This retrospective cohort study used the TriNetX database to identify patients aged 18 years and older with IBD and SARS-CoV-2 infection (IBD-SARS-CoV-2 cohort) during the 2022 and 2023 RSV seasons. A 1:1 propensity score matching was used to compare patients with IBD but no history of SARS-CoV-2 infection (IBD non-SARS-CoV-2 cohort).

**RESULTS:**

In the 2022 IBD-SARS-CoV-2 cohort (mean age: 53.7 ± 17.6 years; 59% female; 77% White), the RSV infection risk was 0.47%, higher than 0.19% in the matched IBD non-SARS-CoV-2 cohort (adjusted odds ratio [aOR]: 2.4; 95% CI: 1.5–3.6). The risk was highest 30–60 days after SARS-CoV-2 infection (aOR: 2.9; 95% CI: 1.7–4.9), particularly in those aged 60 years and older (aOR: 2.5; 95% CI: 1.3–4.5). The use of systemic corticosteroids (aOR: 2.3; 95% CI: 1.1–4.6) or immune-modifying therapies (aOR: 3.9; 95% CI: 2–7.1) was associated with higher RSV infection risk. Similar trends were observed during the 2023 RSV season, with no significant differences in RSV-related hospitalizations.

**DISCUSSION:**

Adults with IBD have a higher risk of RSV infection after SARS-CoV-2 infection, particularly those receiving steroids or immune therapies. SARS-CoV-2 infection may have contributed to the recent RSV infection surge in this population, warranting further research.

## Graphical abstract

**Figure:**
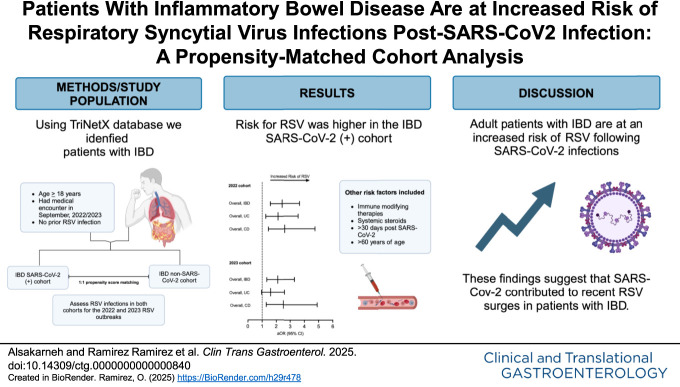

## INTRODUCTION

Respiratory syncytial virus (RSV) is a respiratory virus that typically presents with mild symptoms but can lead to severe disease requiring hospitalization (1). Peak infection rates occur between late fall and early spring in temperate regions, while tropical areas may experience year-round cases. While individuals of all ages are susceptible to infection, infants younger than 1 year and older adults with chronic conditions, such as cardiopulmonary diseases and immunocompromised states, are at a higher risk of severe infections that may require hospitalization (2).

The annual incidence of RSV infection in adults is estimated to be approximately 1.5 million cases. However, this number is likely underestimated owing to the lack of routine testing. Among older adults who are hospitalized with RSV infection, the overall in-hospital mortality rate is 5.6%. This rate is higher for those aged 75 years and older (6.1%) than for those aged 60 to 74 years (4.6%) (3–5). By contrast, adults aged 18–49 years have lower mortality rates, ranging from 0.1 to 0.3 per 100,000 population. Nonetheless, morbidity remains significant in this age group, with hospitalization rates ranging from 8.6 to 13.1 per 100,000 adults (6,7). It is important to note that RSV infections and hospitalizations are prevalent among immunosuppressed populations, where both morbidity and mortality rates are higher than in nonimmunosuppressed individuals (8).

Before the COVID-19 pandemic, RSV followed a predictable seasonal pattern. Initially, the overall incidence of other common respiratory viruses, including RSV, decreased during the pandemic (9). Studies have proposed that this phenomenon is mostly due to the aggressive implementation of nonpharmaceutical interventions (NPIs) such as the use of masks and hand hygiene (4). After this decrease, there has been at least 3 years of higher-than-normal incidence of RSV infection. (10,11). Several studies suggest that decreased viral immunity due to a lack of RSV exposure, referred to as immunity debt, has played an important role in this current situation. However, this concept does not fully explain why surges in RSV infection continue despite liberalization of NPIs leading to other hypotheses, including the idea of immunity theft, which states that COVID-19-induced immune dysregulation may increase the risk of RSV infections (12,13). Previous studies, especially in the pediatric population, have shown an increased risk (RSV) of infection after severe acute respiratory syndrome (SARS) coronavirus 2 (CoV-2) infection (14–17).

Whether there is an increased risk of RSV infection after SARS-CoV-2 infection in adult patients with inflammatory bowel disease (IBD) remains unknown. The primary aim of our study was to examine the risk of RSV infection in patients with IBD diagnosed with a recent SARS-CoV-2 infection compared with those without a diagnosis of SARS-CoV-2 infection. We hypothesized that patients with IBD and a recent diagnosis of SARS-CoV-2 infection would be at an increased risk of RSV infection compared with IBD non-SARS-CoV-2 controls.

## METHODS

### Database

We conducted a retrospective cohort study using the TriNetX (Cambridge, MA) analytics network platform. TriNetX is a multi-institutional global federated research network that contains deidentified data of more than 105 million patients within 61 healthcare organizations across the United States (18). The TriNetX web portal can be used by end-users to send queries to the Advanced Analytics Platform to perform cohort selection, propensity score matching (PSM), and time trend analysis to compare outcomes between cohorts. The deidentification process is determined and performed at the network level by a qualified expert, as defined in the Health Insurance Portability and Accountability Act Privacy Rule. Rigorous quality assurance was achieved at the time of extraction from electronic health records (EHRs) in a systemic and standardized format before inclusion in the database. This study was exempted from the IRB because it involved publicly available deidentified data based on the recommendations of the National Human Research Protection Advisory Committee policy (19). TriNetX has previously been used and validated to study SARS-CoV-2 infections in the United States (20–22). This study followed the Strengthening the Reporting of Observational Studies in Epidemiology reporting guidelines (23).

### Study participants and cohort

A real-time search and analysis of the US Collaborative Network on the TriNetX platform was conducted to examine the association between prior SARS-CoV-2 infection and RSV infection in patients with IBD. The IBD cohort included adults 18 years and older who had *International Classification of Disease, 10th Revision, Clinical Modification* (ICD-10-CM) codes in their EHR for ulcerative colitis (UC) (K51*) or Crohn's disease (CD) (K52*) plus RxNorm codes for at least one of the IBD-related medications: mesalamine, sulfasalazine, balsalazide, olsalazine, azathioprine, mercaptopurine, methotrexate, infliximab, adalimumab, certolizumab pegol, golimumab, vedolizumab, ustekinumab, tofacitinib, oral prednisone, or budesonide. This case definition was validated in a previous study (24) The IBD study population comprised patients who had seen a medical provider during September 2022; this was considered the index event and had no prior RSV infection. This date was selected based on the RSV season of 2022, which peaked from October to December according to data from the National Respiratory and Enteric Virus Surveillance System (10). We additionally included the events after this period from 91 to 180 days after the index date as a secondary analysis.

**Figure 1.**
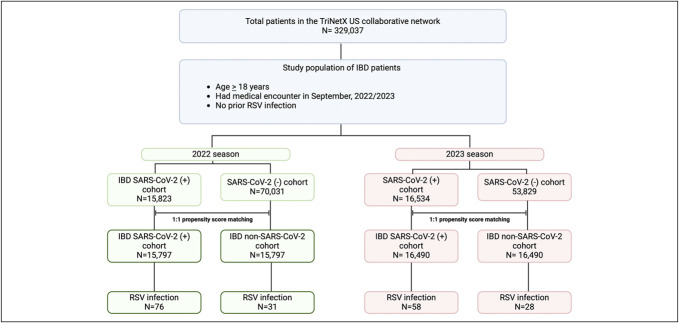
Cohort selection flowchart for assessing RSV infection risk among IBD patients with and without prior SARS-CoV-2 infection across 2022 and 2023 seasons. aOR, adjusted odds ratio; CD, Crohn's disease; CI, confidence interval; IBD, inflammatory bowel disease; RSV, respiratory syncytial virus; SARS-CoV-2, severe acute respiratory syndrome coronavirus 2; UC, ulcerative colitis.

The status of RSV infection was based on 12 laboratory test codes and 3 disease clinical diagnosis codes (See Supplemental Table 1, http://links.lww.com/CTG/B301). The definition used in our analysis has been validated in prior published studies (17) The study population was then divided into 2 IBD cohorts: (i) IBD-SARS-CoV-2 cohort—patients who contracted a recent SARS-CoV-2 infection within the same calendar year 2022 before the index date—and (ii) IBD non-SARS-CoV-2 cohort—patients who had no documented SARS-CoV-2 infection before the index date (Figure 1). SARS-CoV-2 infections were identified by a positive COVID-19-specific laboratory test (LOINC 9088) or ICD-10-CM code for COVID-19 (U07.1). This case definition was validated in previous studies in the United States (17). COVID-19 vaccination status was reported in all patients who received the vaccine before the index date.

For the purpose of this analysis, we included patients who had documented SARS-CoV-2 infection within the calendar year preceding the RSV season, specifically from January 1st to August 30th, 2022. Accordingly, patients with SARS-CoV-2 infection occurring in February 2022 were included in the 2022 analysis, whereas those with infections earlier in 2021 were excluded from both the 2022 and 2023 analyses. This approach ensures that only patients with recent SARS-CoV-2 infection are considered, thereby addressing potential concerns about the impact of earlier infections on RSV susceptibility. Patients with prior RSV diagnosis were excluded from the study.

### Covariates

The covariates and their standardized names, codes, and data types used in this study are listed in Table 1. The IBD-SARS-CoV-2 and control cohorts were created using the following 1:1 PSM variables: (i) demographics (age, sex, race, and ethnicity), (ii) social determinants of adverse health outcomes, (iii) behavioral factors (tobacco smoking and alcohol abuse), and (iv) comorbidities related to RSV morbidity and mortality (chronic kidney disease (CKD), diabetes, and lung disease).

**Table 1.** Baseline characteristics of patients in the IBD-SARS-CoV-2 and IBD non-SARS-CoV-2 control cohorts before and after propensity score matching in 2022

	Before propensity score matching			After propensity score matching		
	IBD-SARS-CoV-2 (n = 15,823)	IBD non-SARS-CoV-2 (n = 70,031)	*P* value	IBD-SARS-CoV-2 (n = 15,797)	IBD non-SARS-CoV-2 (n = 15,797)	*P* value
Demographics						
Age (yr), mean ± SD	53.7 ± 17.6	53.7 ± 18.5	0.98	53.7 ± 17.6	53.9 ± 17.7	0.3559
Female	9,485 (59.9%)	38,593 (55.2%)	<0.0001	9,466 (59.9%)	9,514 (60.2%)	0.58
BMI	29 ± 6.94	28.1 ± 6.65	<0.0001	29 ± 6.93	29.1 ± 7.07	0.3041
Race						
White	12,183 (77.0%)	53,394 (76.2%)	0.044	12,165 (77.1%)	12,430 (78.7%)	0.0003
African American	1,453 (9.2%)	5,268 (7.5%)	<0.0001	1,448 (9.2%)	1,326 (8.4%)	0.0153
Hispanic	832 (5.1%)	2,723 (3.8%)	<0.0001	826 (5.1%)	758 (4.6%)	0.079
Comorbidities						
Hypertension	8,387 (53.1%)	29,304 (41.8%)	<0.0001	8,361 (52.9%)	8,382 (53.1%)	0.8129
Diabetes mellitus	3,939 (24.9%)	11,680(16.7%)	<0.0001	3,913 (24.7%)	3,830 (24.2%)	0.2777
Obesity	5,963 (37%)	16,873 (24%)	<0.0001	5,932 (36.6%)	5,974 (37%)	0.6283
Neoplasms	8,134 (51.6%)	29,508 (42.1%)	<0.0001	8,109 (51.3%)	8,198 (51.8%)	0.3164
Nicotine dependence	2,578 (15.9%)	9,471 (13.3%)	<0.0001	2,572 (15.8%)	2,613 (16.1%)	0.5344
Chronic lower respiratory diseases	6,226 (39.3%)	17,617(25.1%)	<0.0001	6,202 (39.2%)	6,292 (39.8%)	0.3004
Ischemic heart disease	3,665 (22.6%)	10,763 (15.1%)	<0.0001	3,640 (22.5%)	3,541 (21.8%)	0.63
Heart failure	2,157 (13.3%)	5,433 (7.6%)	<0.0001	2,135 (13.1%)	2,035 (12.5%)	0.0971
Chronic kidney disease	2,768 (17.4%)	7,969 (11.3%)	<0.0001	2,746 (17.3%)	2,596 (16.4%)	0.0244
Alcohol related diseases	767 (4.7%)	2,522 (3.5%)	<0.0001	761 (4.7%)	705 (4.3%)	0.1344
HIV	146 (0.9%)	417 (0.6%)	<0.0001	144 (0.9%)	122 (0.7%)	0.2464
Liver or kidney transplant	663 (4.2%)	1,409 (2%)	<0.0001	640 (4.1%)	559 (3.6%)	0.22
Stem cell transplant	98 (0.6%)	268 (0.4%)	<0.0001	96 (0.6%)	89 (0.6%)	0.6057

BMI, body mass index; HIV, human immunodeficiency virus; IBD, inflammatory bowel disease; RSV, respiratory syncytial virus; SARS-CoV-2, severe acute respiratory syndrome coronavirus 2.

### Study aims and outcomes

The primary aim of this study was to examine whether prior SARS-CoV-2 infection during 2022 was associated with an increased risk of RSV infection among patients with IBD. The outcomes were followed through 9/1/2022–12/31/2022. These dates were chosen because the 2022 RSV season peaked during these dates. The TriNetX database allows the creation of temporal associations between different groups within a cohort. This functionality allowed us to identify patients with RSV infection after COVID-19 infection.

The secondary aims were prespecified as follows:

1. Evaluate the risk of RSV infection after COVID-19 hospitalization in the IBD-SARS-CoV-2 cohort compared with the IBD control cohort. Hospitalized patients were required to have a Current Procedural Terminology code for Hospital Inpatient Services (1013659), which occurred within 3 weeks after the diagnosis of SARS-CoV-2 infection. The 3-week period was chosen because most of the patients with COVID-19 are hospitalized within the first 21 days of symptom onset. This definition has also been used in one of our previous studies (25–27).
2. Evaluation of the risk of hospitalization due to RSV infection in the IBD-SARS-CoV-2 and IBD control cohorts.
3. Evaluate the risk of RSV infection during 3 time intervals after the index date between (<30 days, 30–90 days, and 91–180 days after the index date) the IBD-SARS-CoV-2 and IBD control cohorts.
4. Evaluate the effect of comorbidities on risk of RSV infection between the IBD-SARS-CoV-2 and control cohorts. Subgroup analyses were performed based on common comorbidities including diabetes mellitus (DM), heart disease (ischemic heart disease and heart failure), and chronic lower respiratory conditions associated with an increased risk of RSV infection.
5. Evaluate the risk of RSV infections for different age groups and sexes between IBD-SARS-CoV-2 and IBD control cohorts. The age groups were divided into 18–49 years, 50–64 years, and ≥65 years. The cohorts were stratified into male and female. Self-reported sex (female, male) was used as reported in the TriNetX database.
6. Assess the impact of IBD medications on the risk of RSV infection in the IBD-SARS-CoV-2 and IBD control cohorts. IBD medications were divided into those that have been linked to a higher risk of infection. Subgroups were stratified into patients taking (i) anti-TNF agents, (ii) thiopurines or methotrexate, (iii) vedolizumab or ustekinumab, (iv) systemic steroids, and (v) composite of all immune-modifying therapies (steroids, anti-TNF, janus kinase (JAK) inhibitors, azathioprine, and methotrexate). The risk of RSV infection was also assessed in patients who were initiated on prednisone or methylprednisolone within 3 months before the index date between the IBD-SARS-CoV-2 and IBD control cohorts.
7. Evaluate the risk of RSV infection between IBD-SARS-CoV-2 and IBD non-SARS-CoV-2 cohorts. The non-IBD cohort included patients without an ICD-10-CM code for UC or CD. We also performed subgroup analyses of patients with UC and CD.
8. We replicated all analyses to 2023 with the index date being September 2023. The study dates were 9/10/2023–12/31/2023 during the peak RSV season. In addition, we included patients with SARS-CoV-2 infection during the 2023 season before the index date. Those with a previous RSV infection or SARS-CoV-2 infection before 2023 were excluded.

### Statistical analyses

All statistical tests were performed using built-in functions of the TriNetX Advanced Analytics Platform. The characteristics of the 2 groups are presented as mean ± SD or frequency and proportion. One-to-one (1:1) PSM was performed to balance the covariate distribution between the 2 groups. The TriNetX platform uses input matrices of user-identified covariates to conduct a logistic regression analysis to obtain propensity scores for all individual subjects. The propensity scores generated were used to match patients using greedy nearest-neighbor algorithms with a caliper width of 0.1 pooled standard deviations. TriNetX randomizes the order of rows to eliminate bias resulting from the nearest-neighbor algorithms. After propensity matching, the risk of each outcome was calculated and expressed as adjusted odds ratios (aORs) with 95% confidence intervals (CIs). Statistical significance was set at *P* < 0.05.

## RESULTS

### Characteristics of the study population

In the 2022 RSV season, 70,031 patients were included in the IBD non-SARS-CoV-2 cohort and 15,823 in the IBD-SARS-CoV-2 cohort. After PSM, 15,797 patients were analyzed in each group. In the 2023 RSV season, 53,829 patients were included in the IBD non-SARS-CoV-2 cohort and 16,534 in the IBD-SARS-CoV-2 cohort. After PSM, 16,490 patients were included in each cohort. All demographic parameters and comorbid conditions in the IBD-SARS-CoV-2 cohort and IBD non-SARS-CoV-2 cohorts during the 2022 and 2023 RSV seasons before and after PSM are summarized in Tables 1 and 2.

**Table 2.** Baseline characteristics of patients in the IBD-SARS-CoV-2 and IBD non-SARS-CoV-2 control cohorts before and after propensity score matching in 2023

	Before propensity score matching			After propensity score matching		
	IBD-SARS-CoV-2 (n = 16,534)	IBD non-SARS-CoV-2 (n = 53,829)	*P* value	IBD-SARS-CoV-2 (n = 16,490)	IBD non-SARS-CoV-2 (n = 16,490)	*P* value
Demographics						
Age (yr), mean ± SD	55.8 ± 17.4	54.3 ± 18.4	<0.0001	55.8 ± 17.4	56.1 ± 17.5	0.066
Female	9,834 (59.4%)	29,133 (54.1%)	<0.0001	9,798 (59.4%)	9,905 (60%)	0.23
BMI	28.8 ± 6.77	28.1 ± 6.55	<0.0001	28.8 ± 6.75	29 ± 6.95	0.0543
Race						
White	12,807 (77.4%)	41,236 (76.6%)	0.0231	12,780 (77.5%)	13,058 (79.2%)	0.0002
African American	1,501 (9.1%)	4,538 (8.4%)	0.0093	1,494 (9.1%)	1,415 (8.6%)	0.1250
Hispanic	877 (5.2%)	2,224 (4%)	<0.0001	862 (5.1%)	737 (4.4%)	0.0014
Comorbidities						
Hypertension	9,319 (56.4%)	23,774 (44.2%)	<0.0001	9,274 (56.2%)	9,349 (56.7%)	0.4049
Diabetes mellitus	4,363 (26.4%)	9,843 (18.3%)	<0.0001	4,325 (26.2%)	4,255 (25.8%)	0.3796
Obesity	6,530(38.7%)	14,322 (26%)	<0.0001	6,454 (38.4%)	6,532 (38.9%)	0.3821
Neoplasms	9,158 (55.4%)	23,752 (44.1%)	<0.0001	9,117 (55.3%)	9,251 (56.1%)	0.1374
Nicotine dependence	2,719 (16.1%)	8,076 (14.7%)	<0.0001	2,700 (16.1%)	2,674 (16%)	0.6988
Chronic lower respiratory diseases	6,878 (41.6%)	14,511 (27%)	<0.0001	6,838 (41.5%)	6,948 (42.1%)	0.2194
Ischemic heart disease	4,168 (24.7%)	9,111 (16.6%)	<0.0001	4,102 (24.4%)	3,993 (23.8%)	0.1643
Heart failure	2,444 (14.5%)	4,597 (8.3%)	<0.0001	2,387 (14.2%)	2,287 (13.6%)	0.1149
Chronic kidney disease	3,142 (19%)	6,769 (12.6%)	<0.0001	3,106 (18.8%)	3,010 (18.3%)	0.1738
Alcohol related diseases	878 (5.2%)	2,192 (4%)	<0.0001	862 (5.1%)	828(4.9%)	0.3961
HIV	131 (0.8%)	323 (0.6%)	0.0069	130 (0.8%)	102 (0.6%)	0.0651
Liver or kidney transplant	685 (4.2%)	1,081 (2%)	<0.0001	642 (3.9%)	599 (3.6%)	0.4485
Stem cell transplant	115 (0.7%)	231 (0.4%)	<0.0001	114 (0.7%)	100 (0.6%)	0.3370

BMI, body mass index; IBD, inflammatory bowel disease; HIV, human immunodeficiency virus; SARS-CoV-2, severe acute respiratory syndrome coronavirus 2.

### Risk of RSV infection after SARS-CoV-2 infection in the 2022 RSV season

**Figure 2.**
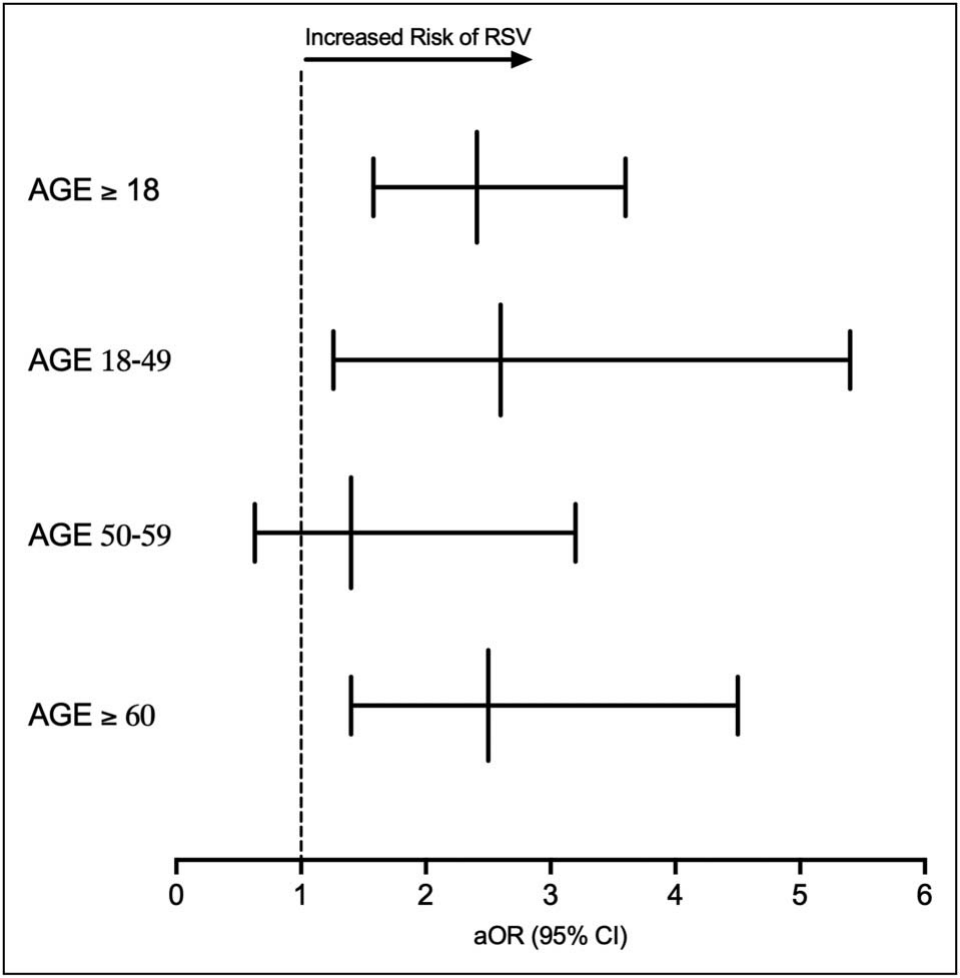
Risk of RSV infection in the IBD-severe acute respiratory syndrome coronavirus 2 cohort compared with the IBD control cohort stratified by age group during 2022 RSV season. aOR, adjusted odds ratio; CI, confidence interval; IBD, inflammatory bowel disease; RSV, respiratory syncytial virus.

After PSM, we identified 76 patients (0.47%) who developed RSV infection in the IBD-SARS-CoV-2 cohort and 31 (0.19%) in the IBD non-SARS-CoV-2 control cohort, representing a significantly increased risk of RSV infection in the IBD-SARS-CoV-2 cohort compared with the control group (aOR, 2.41; 95% CI, 1.583–3.665). There was no significant difference in the risk of RSV infection (aOR, 1.006; 95% CI, 0.419–2.418) within 0–29 days after diagnosis between the 2 cohorts. However, the risk was higher in the 30–90-day (aOR, 2.9; 95% CI, 1.704–4.981) and 81–190-day (aOR, 2.6; 95% CI, 1.456–4.482) intervals. The IBD-SARS-CoV-2 cohort patients aged 18–49 years (aOR, 2.6; 95% CI, 1.262–5.437) and 60 years and older (aOR, 2.5; 95% CI, 1.366–4.544) (Figure 2) were at an increased risk of RSV infection compared with the same age group and sex-matched IBD non-SARS-CoV-2 control.

Comorbidity analysis indicated that patients with chronic lung disease (CLD) (aOR, 2.44; 95% CI, 1.483–4.015), CKD (aOR, 2.22; 95% CI, 1.087–4.544), and DM (aOR, 3.05; 95% CI, 1.488–6.242) were at an increased risk of RSV infection in the IBD-SARS-CoV-2 cohort compared with the control group. Medication analysis showed that patients receiving systemic steroids (aOR, 2.31; 95% CI, 1.132–4.691) and immune-modifying therapies (aOR, 3.9; 95% CI, 2.099–7.126) were also at increased risk of RSV infection. However, there was no significant difference in the risk of RSV infection between patients on thiopurines or methotrexate (aOR, 1.72; 95% CI, 0.787–3.763) and anti-TNF alone (aOR, 1.4; 95% CI, 0.626–3.179) when compared with the IBD non-SARS-CoV-2 control cohort (Table 3).

**Table 3.** Risk of RSV in patients with IBD in the IBD-SARS-CoV-2 cohort and IBD non-SARS-CoV-2 cohort (control group) in the 2022 RSV season after propensity score matching

Analyses	IBD SARS-CoV-2 (n = 15,797) N of cases/total cohort, (%^a^)	IBD control (n = 15,797) N of cases/total cohort, (%^a^)	aOR, 95% CI	*P* Value
IBD	76/15,797 (0.48)	31/15,797 (0.19)	2.41 (1.583, 3.665)	<0.0001
Time interval				
0–29 d	10/15,797 (0.06)	10/15,797 (0.06)	1.006 (0.419, 2.418)	0.9
30–90 d	52/15,797 (0.33)	18/15,797 (0.11)	2.9 (1.704, 4.981)	<0.0001
81–190 d	43/15,797 (0.27)	17/15,797 (0.11)	2.6 (1.456, 4.482)	0.0001
Hospitalization status				
Inpatient care	15/2,147 (0.7)	10/2,147 (0.46)	1.526 (0.684, 3.404)	0.2983
Outpatient care	61/13,555 (0.45)	28/13,555 (0.21)	2.2 (1.402, 3.435)	0.0004
Age groups				
≥18 years old	76/15,797 (0.48)	31/15,797 (0.19)	2.41 (1.583, 3.665)	<0.0001
18–49 years old	26/5,934 (0.44)	10/5,934 (0.17)	2.6 (1.262, 5.437)	0.0072
50–59 years old	14/2,861 (0.50)	10/2,861 (0.35)	1.4 (0.626, 3.183)	0.404
≥60 years old	37/7,084 (0.52)	15/7,084 (0.21)	2.5 (1.366, 4.544)	0.002
Sex^b^				
Male	26/5,569 (0.47)	10/5,569 (0.18)	2.623 (1.264, 5.444)	0.0072
Female	48/9,342 (0.51)	21/9,342 (0.22)	2.309 (1.382, 3.859)	0.001
Comorbidities				
CLD	53/7,480 (0.71)	22/7,480 (0.29)	2.44 (1.483, 4.015)	0.0003
CVD^c^	31/4,868 (0.64)	20/4,868 (0.41)	1.57 (0.893, 2.758)	0.1139
CKD	24/3,305 (0.73)	11/3,305 (0.33)	2.22 (1.087, 4.544)	0.0247
Nicotine dependence	15/3,375 (0.44)	10/3,375 (0.3)	1.517 (0.681, 3.382)	0.3044
DM	30/4,379 (0.69)	10/4,379 (0.23)	3.05 (1.488, 6.242)	0.0014
Medications				
Thiopurines or methotrexate	17/3,719 (0.46)	10/3,719 (0.27)	1.72 (0.787, 3.763)	0.1688
Anti-TNF	14/4,227 (0.33)	10/4,227 (0.24)	1.4 (0.626, 3.179)	0.4047
Ustekinumab or vedolizumab	10/2,428 (0.41)	10/2,428 (0.41)	1 (0.416, 2.408)	0.9
Steroids	25/3,566 (0.7)	10/3,566 (0.31)	2.31 (1.132, 4.691)	0.0178
All immune-modifying therapies, steroids, anti-TNF, JAKi, AZA, MTX	50/6,752 (0.74)	13/6,752 (0.19)	3.9 (2.099, 7.126)	<0.0001
Mesalamine	25/5,999 (0.42)	10/5,999 (0.17)	2.44 (1.173, 5.086)	0.013

One-to-one (1:1) propensity score matching between the 2 cohorts was performed for demographic variables, comorbidities, and RSV risk factors.

aOR, adjusted odds ratio; AZA, azathioprine; CI, confidence interval; CKD, chronic kidney disease; CLD, chronic lung disease; CVD, cardiovascular disease; DM, diabetes mellitus; IBD, inflammatory bowel disease; MTX, methotrexate; JAKi, janus kinase inhibitor; RSV, respiratory syncytial virus; SARS-CoV-2, severe acute respiratory syndrome coronavirus 2; TNF, tumor necrosis factor.

a The percentages shown in the tables are calculated by dividing the number of cases by the total number of individuals in the respective cohort.

b Patients who were not classified as male or female were not included in the analysis due to database restrictions.

c CVD includes ischemic heart disease and heart failure.

### Risk of RSV infection after SARS-CoV-2 infection in the 2023 RSV season

Fifty-eight patients (0.36%) developed RSV infection in the IBD-SARS-CoV-2 cohort and 28 (0.17%) in the control group. After PSM, the IBD-SARS-CoV-2 cohort had an increased risk of RSV infection (aOR, 2.1; 95% CI, 1.33–3.3) compared with the IBD non-SARS-CoV-2 control group. The risk of RSV infection was higher in the 30–90-day (aOR, 1.95; 95% CI, 1.021–3.715) and 81–190-day (aOR, 2.6; 95% CI, 1.135–4.877) intervals, whereas there was no significant difference in the risk of RSV infection within 0–29 days after diagnosis (aOR, 1.009; 95% CI, 0.42–2.425). Regarding age groups, the risk was higher for patients aged 60 years and older (aOR, 3.6; 95% CI, 1.841–7.029).

Comorbidity analysis showed that patients with CLD (aOR, 3.269; 95% CI, 1.793–5.96) and cardiovascular disease (CVD) (aOR, 2.505; 95% CI, 1.313–4.778) were at an increased risk of RSV infection in the IBD-SARS-CoV-2 cohort compared with controls. Medication analysis showed that patients on immune-modifying therapies (aOR, 4.22; 95% CI, 2.116–8.418) were at increased risk of RSV infection; use of systemic steroids also showed an increase in risk, although it was not significant (aOR, 2.0; 95% CI, 0.9–4.2). However, there was no significant difference in the risk of RSV infection between patients on thiopurines or methotrexate (aOR, 1.012; 95% CI, 0.421–2.434) and anti-TNF (aOR, 1.009; 95% CI, 0.419–2.426) when compared with the IBD non-SARS-CoV-2 control cohort.

**Figure 3.**
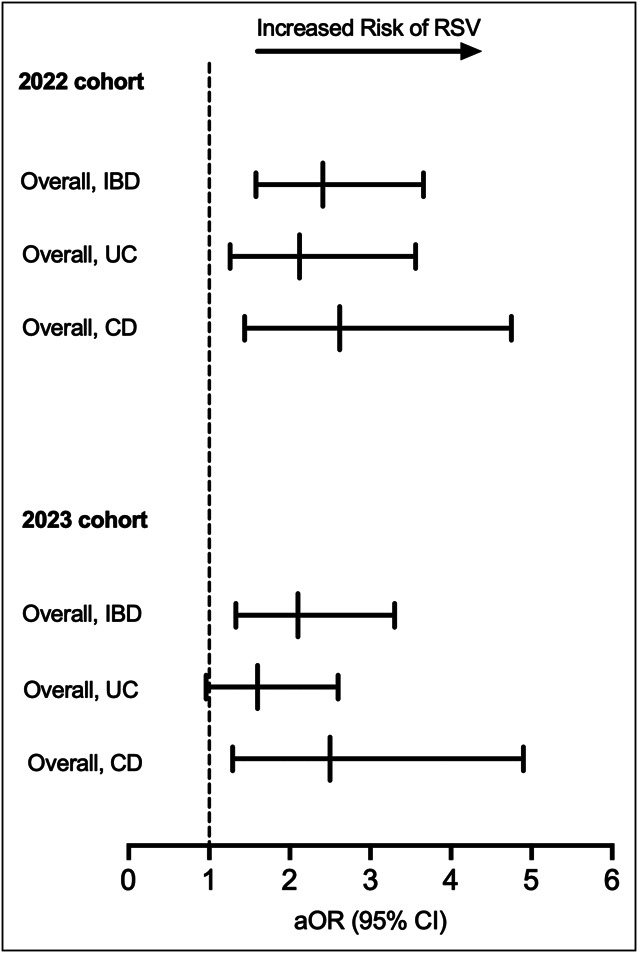
Risk of RSV infection in the IBD-severe acute respiratory syndrome coronavirus 2 cohort compared with the IBD control cohort in 2022 and 2023 RSV seasons. aOR, adjusted odds ratio; CD, Crohn's disease; CI, confidence interval; IBD, inflammatory bowel disease; RSV, respiratory syncytial virus; UC, ulcerative colitis.

After PSM, there was no increased risk of RSV-related hospitalizations during the RSV seasons of 2022 and 2023 (aOR, 0.951; 95% CI, 0.513–1.766 and aOR, 1.4; 95% CI, 0.694–2.862, respectively) (Figure 3). The study also assessed SARS-CoV-2 vaccination status. In the 2022 analysis, COVID-19 vaccination rates were significantly different between groups: 14.4% in the IBD-SARS-CoV-2 cohort vs 9.8% in the IBD non-SARS-CoV-2 control cohort (*P* < 0.001). For 2023, vaccination rates were significantly different between groups: 15.7% in the IBD-SARS-CoV-2 cohort vs 10.8% in the IBD non-SARS-CoV-2 control cohort (*P* < 0.001) (Table 4).

**Table 4.** Risk of RSV in patients with IBD in the IBD-SARS-CoV-2 cohort and IBD non-SARS-CoV-2 cohort (control group) in the 2023 RSV season

Analyses	IBD SARS-CoV-2 (n = 16,490) N of cases/total cohort, (%^a^)	IBD control (n = 16,490) N of cases/total cohort, (%^a^)	aOR, 95% CI	*P* Value
IBD	58/16,490 (0.35)	28/16,490 (0.17)	2.1 (1.33, 3.3)	0.001
Time interval				
0–29 d	10/16,490 (0.06)	10/16,490 (0.06)	1.009 (0.42, 2.425)	0.9
30–90 d	27/16,490 (0.16)	14/16,490 (0.09)	1.95 (1.021, 3.715)	0.0394
81–190 d	33/16,490 (0.20)	13/16,490 (0.08)	2.6 (1.35, 4.877)	0.0028
Hospitalization status				
Inpatient care	17/2,194 (0.78)	10/2,194 (0.45)	1.74 (0.795, 3.81)	0.1602
Outpatient care	41/14,035 (0.29)	22/14,035 (0.16)	1.9 (1.119, 3.156)	0.0154
Age groups				
≥18 years old	58/16,490 (0.35)	28/16,490 (0.17)	2.1 (1.33, 3.3)	0.001
18–49 years old	13/5,740 (0.23)	10/5,740 (0.17)	1.31 (0.575, 2.995)	0.517
50–59 years old	12/2,949 (0.40)	10/2,949 (0.34)	1.21 (0.522, 2.805)	0.65
≥60 years old	39/7,774 (0.50)	11/7,774 (0.14)	3.6 (1.841, 7.029)	<0.0001
Sex^b^				
Male	18/5,676 (0.32)	10/5,676 (0.18)	1.81 (0.837, 3.937)	0.1253
Female	34/9,610 (0.35)	17/9,610 (0.18)	2.025 (1.131, 3.627)	0.0154
Comorbidities				
CLD	45/7,823 (0.58)	14/7,823 (0.18)	3.269 (1.793, 5.96)	<0.0001
CVD^c^	32/5,148 (0.62)	13/5,148 (0.25)	2.505 (1.313, 4.778)	0.0039
CKD	22/3,344 (0.66)	12/3,344 (0.36)	1.86 (0.919, 3.764)	0.0797
Nicotine dependence	19/3,423 (0.56)	10/3,423 (0.29)	1.927 (0.895, 4.15)	0.0881
DM	16/4,598 (0.35)	14/4,598 (0.3)	1.158 (0.565, 2.375)	0.6886
Medications				
Thiopurines or methotrexate	10/3,679 (0.27)	10/3,679 (0.27)	1.012 (0.421, 2.434)	0.9792
Anti-TNF	10/4,135 (0.24)	10/4,135 (0.24)	1.009 (0.419, 2.426)	0.9845
Ustekinumab or vedolizumab	10/2,564 (0.39)	10/2,564 (0.39)	1.002 (0.416, 2.411)	0.99
Steroids	19/3,361 (0.57)	10/3,361 (0.29)	2.006 (0.938, 4.291)	0.0673
All immune modifying therapies, anti-TNF, JAKi, AZA, MTX	42/6,647 (0.63)	10/6,647 (0.15)	4.22 (2.116, 8.418)	< 0.0001
Mesalamine	16/6,071 (0.26)	10/6,071 (0.16)	1.613 (0.731, 3.557)	0.2319

One-to-one (1:1) propensity score matching between the 2 cohorts was performed for demographic variables, comorbidities, and RSV risk factors.

aOR, adjusted odds ratio; AZA, azathioprine; CI confidence interval; CKD, chronic kidney disease; CLD, chronic lung disease; CVD, cardiovascular disease; DM, diabetes mellitus; IBD, inflammatory bowel disease; JAKi, janus kinase inhibitors; MTX, methotrexate; RSV, respiratory syncytial virus; SARS-CoV-2, severe acute respiratory syndrome coronavirus 2; TNF, tumor necrosis factor.

a The percentages shown in the tables are calculated by dividing the number of cases by the total number of individuals in the respective cohort.

b Patients who were not classified as male or female were not included in the analysis due to database restrictions.

c CVD includes ischemic heart disease and heart failure.

## DISCUSSION

To the best of our knowledge, this study is the first to investigate the risk of RSV infection in patients with IBD who had a history of recent SARS-CoV-2 infection. Our findings demonstrate that patients with IBD who have recently experienced SARS-CoV-2 infection are at an increased risk of subsequent RSV infection compared with those without a history of SARS-CoV-2 infection. This increased risk was observed during the RSV seasons in 2022 and 2023, indicating a consistent pattern. Notably, a higher risk was observed in the 30–90-day and 81–190-day postdiagnosis intervals, indicating prolonged susceptibility to RSV infection after SARS-CoV-2 infection.

Our findings align with our previous research, further suggesting that immune dysregulation caused by SARS-CoV-2 infection may have lasting effects, increasing vulnerability to secondary infections, such as reactivation of varicella zoster virus in the form of herpes zoster (27). The proposed mechanisms for this increase in susceptibility include lymphopenia affecting T-lymphocytes, B-lymphocytes, and natural killer cell populations, along with a cytokine-mediated inflammatory milieu. This environment may lead to prolonged activation and subsequent exhaustion of both T and B-lymphocytes, potentially compromising the host's ability to mount an effective immune response against subsequent RSV infection or other secondary infections (17,28,29). A retrospective multicenter study during 2021 and 2022 that included 228,990 children demonstrated a significantly higher risk of RSV infection in those with a COVID-19 infection history compared with those without a history of COVID-19 with a risk ratio of 1.40 (95% CI 1.27 to 1.55) in 2022 and 1.32 (95% CI 1.12 to 1.56) in 2021 (17). A retrospective cohort study examining RSV epidemiology in the years surrounding the COVID-19 pandemic also identified a 3 to fourfold increase in infant hospitalizations, while diagnostic shifts contributed to a doubling of adult outpatient detections compared with prepandemic years (30).

Age-specific analyses showed that an increased risk of RSV infection was present across the various age groups. Specifically, patients aged 60 years and older were at the highest risk in our study, which is consistent with previous literature showing a higher incidence of RSV infection (11,31,32). Those aged 18–49 years also had a higher risk of RSV infection in our study population during the RSV season of 2022. This finding highlights the susceptibility to RSV infection among younger patients with IBD who have recently experienced SARS-CoV-2 infection. Notably, these younger individuals are not currently eligible for RSV vaccination, leaving them particularly vulnerable. Given that this risk was higher, although not statistically significant in the 2023 RSV season, and the absolute risk was small, future cost-effectiveness analyses and prospective studies targeting this age group are necessary to determine whether expanding vaccine eligibility would decrease morbidity and healthcare utilization.

Other than age, there are well-defined comorbidities reported in the literature that are linked to increased risk and severity of RSV infection. These factors include CLD, CVD (including coronary artery disease and chronic heart failure), DM, and CKD (33–36). Our findings indicate that in the 2022 RSV season, patients with CLD, CKD, or DM were at a significantly higher risk of RSV infection in the IBD-SARS-CoV-2 cohort than in the control group, suggesting a synergistic effect related to SARS-CoV-2 infection. In the 2023 RSV season, patients with CLD or CVD had a significantly higher risk of RSV infection in the IBD-SARS-CoV-2 cohort. Patients with CKD or CVD were also at an increased risk, although the difference was not statistically significant. The differences in statistical significance across the years could be partly related to the smaller sample size.

In our current analysis, we did not find an increased risk of RSV-related hospitalizations in either the SARS-CoV-2-exposed or unexposed IBD cohorts. This outcome differs from our earlier research, which included a larger patient population (794 patients with RSV infection) and higher hospitalization rates (45.6%). The discrepancy in these findings likely represents reduced statistical power because of a smaller total number of patients after PSM, limiting our ability to detect meaningful differences in hospitalization risk. Consequently, while we still observed an increased risk of RSV infection, this did not translate into a significant rise in hospitalizations.

In patients with IBD, the use of systemic steroids and anti-TNF inhibitors is associated with a higher risk of pneumococcal and influenza infections. On the other hand, immunomodulators such as thiopurines and methotrexate do not seem to significantly increase the risk of pneumococcal pneumonia (37–39). JAK inhibitors are also associated with a lower antibody response to influenza vaccination, indicating a higher risk of influenza infection (40). Although there are limited studies on other immunosuppressed populations, a recent study found that adults with IBD who contract RSV infection are at an increased risk of hospitalization (2). In our study, subgroup analysis of the use of immune-modifying therapies in our population revealed that the use of systemic steroids and a composite of immune-modifying therapies (steroids, anti-TNF, JAK inhibitors, azathioprine, and methotrexate) represented a higher risk of RSV infection in the IBD-SARS-CoV-2 cohort than in the control group. Individual assessments of each immune-modifying medication did not show a statistically significant difference, most likely because of the smaller sample size.

Our findings have several important clinical implications. Given the increased risk of RSV infection after SARS-CoV-2 infection in patients with IBD, healthcare providers must consider preventive strategies, including RSV vaccination, and for eligible patients, the Advisory Committee on Immunization Practices now recommends RSV vaccination for all adults aged 75 years and older. In addition, adults aged 60–74 years who are at an increased risk of severe disease are also recommended to receive the vaccine. A European consensus by leading German societies recommends the use of RSV vaccination in persons aged 60 years or older, as well as in adults of any age with severe pulmonary or cardiovascular preexisting conditions or significant immune compromise (defined as patients on systemic glucocorticoids, after solid transplantation or significant cytopenias) (41,42). Our data suggest that a broader vaccination strategy might also benefit younger patients with IBD, particularly those with additional comorbidities or on immune-modifying therapy, although more evidence is warranted.

This study provides new robust data on patients with IBD about the risk of RSV infection after SARS-CoV-2 infection. One of the notable strengths of this study is the large and diverse sample size, which was made possible by using a multicenter database, allowing for the generalization of results. PSM was used to minimize the observed confounders related to RSV risk factors between the treated and control groups, making the groups more comparable. In addition, secondary analyses provided a broader understanding of the risk factors of RSV infection and outcomes related to infection in our population with IBD and SARS-CoV-2 infection.

Our study has several limitations that should be considered, which could have implications for future research. The retrospective design and reliance on EHRs may introduce information bias and limit the ability to capture all relevant variables, including social and economic factors that could influence outcomes. Despite PSM, there may still be residual confounding from unmeasured variables. In addition, the sample size can decrease after matching, which could reduce statistical power, particularly in subgroup analyses with smaller sample sizes. Although our cohort showed an increased relative risk of RSV infection, the absolute incidence was small, making it difficult to interpret the observed association. Furthermore, the lack of an increased risk of RSV-related hospitalizations highlights the need for further research to clarify the clinical relevance of these findings.

In addition, the database does not provide information on the frequency of testing. Consequently, the observed incidence of RSV infection may be influenced by variations in diagnostic practices between inpatient and outpatient settings rather than representing actual differences in infection rates. It is important to recognize that NPI used to prevent COVID-19 may have also affected the incidence of RSV infection. As a result, patients without COVID-19 may have experienced lower rates of RSV, making it a relevant confounding factor to consider.

In conclusion, our study highlights that adult patients with IBD are at an increased risk of RSV infection after SARS-CoV-2 infection. These findings suggest that SARS-CoV-2 infection contributed to the 2022 and 2023 surge of RSV cases in patients with IBD, especially those on systemic steroid or immune-modifying therapies. Further research is warranted to explore the underlying mechanisms driving increased susceptibility to RSV infection after SARS-CoV-2 infection.

## CONFLICTS OF INTEREST

**Guarantor of the article:** Freddy Caldera, DO, MS, PhD, FACG.

**Specific author contributions:** S.A.: study concept and design, acquisition of data, analysis and interpretation of data, drafting of manuscript and critical revision of manuscript. O.R.R.: analysis and interpretation of data, drafting of manuscript and critical revision of manuscript. M.S.H.: critical revision of manuscript. J.G.H.: critical review of the manuscript. F.A.F.: critical revision of manuscript. F.C.: study concept and design, analysis and interpretation of data, drafting of manuscript, and critical revision of manuscript.

**Financial support:** None to report.

**Potential competing interests:** Dr. Freddy Caldera has received research support from Takeda Pharmaceuticals, Janssen and Novavax. He has been a consultant for Takeda, Arena Pharmaceuticals, GSK, and Celgene. Dr. Hashash served on an Advisory Board for Bristol Myers Squibb. Dr. Farraye is a consultant for Avalo Therapeutics, BMS, Braintree Labs, Fresenius Kabi, GI Reviewers, GSK, Iterative Health, Janssen, Pfizer, Sandoz Immunology, Sebela and Viatris. He sits on a DSMB for Lilly Pharmaceuticals Dr. Hayney is a consultant for GSK Vaccines and has received research support from Takeda Pharmaceuticals, Novavax, and Dynavax.

**IRB statement:** This study was exempted from the IRB as it involved publicly available deidentified data based on the recommendations of the National Human Research Protection Advisory Committee policy.

**Study Highlights**

**WHAT IS KNOWN**

✓ Patients with inflammatory bowel disease have an increased susceptibility to infections.
✓ Respiratory syncytial virus (RSV) typically causes mild symptoms but can lead to severe disease in high-risk groups.
✓ Before the COVID-19 pandemic, RSV infection had a predictable seasonal pattern, now disrupted by 3 years of increased incidence.

**WHAT IS NEW HERE**

✓ Prior severe acute respiratory syndrome coronavirus 2 infection is associated with a higher risk of RSV infection.
✓ The risk is higher within 30–60 days after infection and among those receiving immune-modifying therapies.
✓ These findings suggest that severe acute respiratory syndrome coronavirus 2 infection increases susceptibility to RSV infection in patients with inflammatory bowel disease.

## Supplementary Material

SUPPLEMENTARY MATERIAL
