# Supplementary material for: Patients With Inflammatory Bowel Disease Are at Increased Risk of Respiratory Syncytial Virus Infections After Severe Acute Respiratory Syndrome Coronavirus 2 Infection: A Propensity-Matched Cohort Analysis
Source: Clin Transl Gastroenterol. 2025 Mar 27;16(5):e00840. doi: 10.14309/ctg.0000000000000840 (PMC12101911; doi:10.14309/ctg.0000000000000840)
Supplement: SUPPLEMENTARY MATERIAL [file ct9-16-e00840-s001.docx]

Supplementary Table 1

| The status of RSV infection was based on 12 lab test codes and 3 disease clinical diagnosis codes (Logical Observation Identifiers Names and Codes (LOINC) |
| --- |
| Respiratory syncytial virus RNA [Presence] in Specimen by NAA with probe detection (Logical Observation Identifiers Names and Codes (LOINC) code: 40988-8) |
| Respiratory syncytial virus RNA [Presence] in Nasopharynx by NAA with probe detection (LOINC code 76089-2) |
| Respiratory syncytial virus RNA [Presence] in Nasopharynx by NAA with non-probe detection (LOINC code 82176-9) |
| Respiratory syncytial virus RNA [Presence] in Respiratory specimen by NAA with probe detection (LOINC code 92131-2) |
| Respiratory syncytial virus RNA [Presence] in Isolate by NAA with probe detection (LOINC code 60271-4) |
| Respiratory syncytial virus RNA [Presence] in Upper respiratory specimen by NAA with probe detection (LOINC code 85479- 4) |
| Respiratory syncytial virus Ag [Presence] in Specimen (LOINC code 31950-9) |
| Respiratory syncytial virus Ag [Presence] in Nasopharynx by Rapid immunoassay (LOINC code 72885-7) |
| Respiratory syncytial virus Ag [Presence] in Nose (LOINC code 33045-6) |
| Respiratory syncytial virus Ab [Presence] in Serum (LOINC code 33390-6) |
| Respiratory syncytial virus Ag [Presence] in Specimen by Immunoassay (LOINC code 5876-8 |
| Respiratory syncytial virus Ag [Presence] in Specimen by Immunofluorescence (LOINC code 5877-6) |
| ICD 10 Codes |
| Respiratory syncytial virus as the cause of diseases classified elsewhere (The International Classification of Diseases, Tenth Revision (ICD-10) code: B97.4) |
| Respiratory syncytial virus pneumonia (ICD-10 code: J12.1) |
| Acute bronchiolitis due to respiratory syncytial virus (ICD-10 code: J21.0) |
